# Left atrial appendage occlusion and radiofrequency ablation in a patient with atrial fibrillation and dextrocardia: a case report

**DOI:** 10.3389/fcvm.2024.1525387

**Published:** 2025-01-15

**Authors:** Zengfu Zhang, Xiaohong Fu, Min Guo, Jia Gao, Rui Wang

**Affiliations:** ^1^ The First Clinical Medical College of Shanxi Medical University, Taiyuan, Shanxi, China; ^2^ Department of Cardiology, First Hospital of Shanxi Medical University, Taiyuan, Shanxi, China

**Keywords:** dextrocardia, atrial fibrillation, left atrial appendage occlusion, radiofrequency ablation, intracardiac echocardiography

## Abstract

**Background:**

Dextrocardia is a rare congenital condition, affecting approximately 1 in 10,000–12,000 individuals. When combined with atrial fibrillation (AF), it becomes even rarer. “One-stop” surgery, including combined radiofrequency ablation (RFA) and left atrial appendage occlusion (LAAO), has become a common clinical treatment for patients with AF who develop cerebral infarction despite regular oral anticoagulants. To date, no cases have been reported of patients with AF and dextrocardia undergoing the “one-stop” procedure, making this surgery particularly challenging.

**Case presentation:**

An 85-year-old dextrocardia male with total visceral inversion and persistent AF developed cerebral infarction despite regular oral anticoagulation therapy. He was referred to our hospital for RFA of AF and LAAO. The procedure was successfully performed using a three-dimensional electroanatomical mapping system (Carto3, Biosense Webster, Diamond Bar, CA, USA), intracardiac echocardiography (ICE), and x-ray, with no complications.

**Conclusion:**

This is the first reported case of a “one-stop” surgery for dextrocardia with AF. This procedure is safe and feasible with the assistance of advanced technologies such as ICE and the VIZIGO bidirectional adjustable bent sheath.

## Introduction

Dextrocardia is a congenital malposition of the heart that occurs in approximately 1 in 10,000–12,000 individuals (1). Radiofrequency ablation (RFA) has recently become widely used to treat atrial fibrillation (AF). Additionally, left atrial appendage occlusion (LAAO) is increasingly accepted to prevent cerebral infarction and bleeding complications associated with oral anticoagulant therapy (2). While there have been reports of RFA and LAAO procedures performed in patients with dextrocardia (3–5), there have been no reports of the simultaneous “one-stop” surgery (combined RFA and LAAO) in this patient population.

## Case description

**Figure 1:**
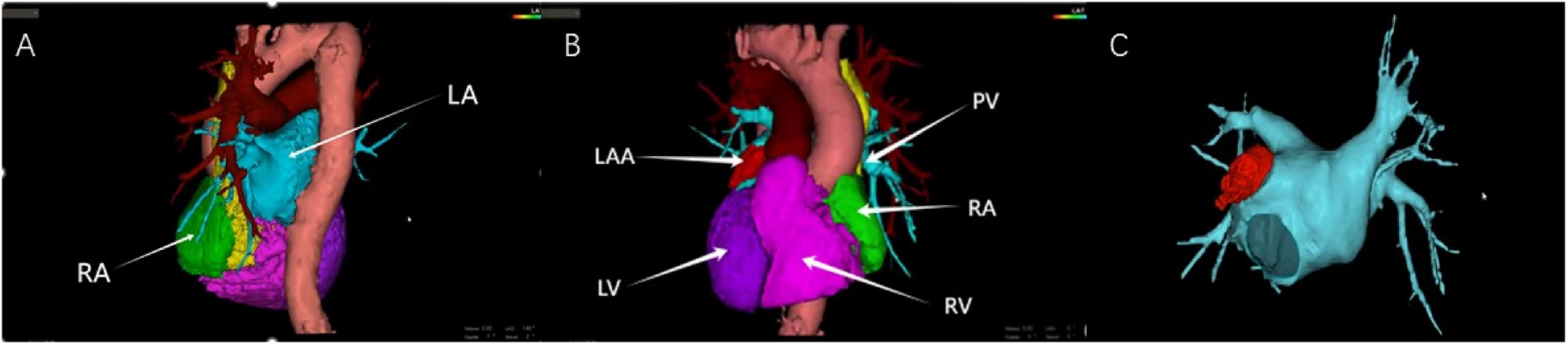
**(A)** Computed tomography (CT) three-dimensional (3D) reconstruction of the heart (RA, right atrium; LA, left atrium); **(B)** CT 3D reconstruction of the heart (LAA, left atrial appendage, LV, left ventricle, PV, pulmonary vein, RA, right atrium, and RV, right ventricle); **(C)** CT 3D reconstruction of the left atrium.

**Figure 2:**
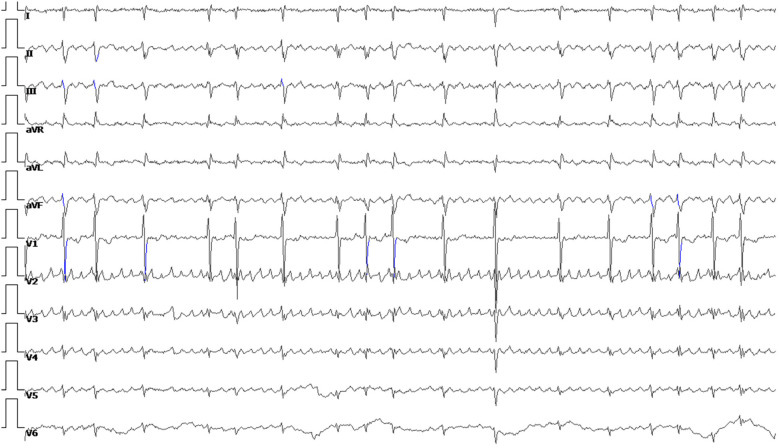
Electrocardiogram of a patient with atrial fibrillation.

An 85-year-old male patient with dextrocardia (Figure 1) was hospitalized 4 years ago due to cerebral infarction, during which an electrocardiogram revealed AF. He was later referred to our hospital after recurrent cerebral infarctions despite regular oral anticoagulant therapy (rivaroxaban 15 mg/day). A 24 hour Holter electrocardiogram confirmed persistent AF (Figure 2). The patient's CHA2DS2-VASc score was 5, and his HAS-BLED score was 3, both indicating the need for “one-stop” surgery (6, 7). After explaining the risks and benefits of the procedure, the patient agreed to proceed with treatment after signing the informed consent form.

**Figure 3:**
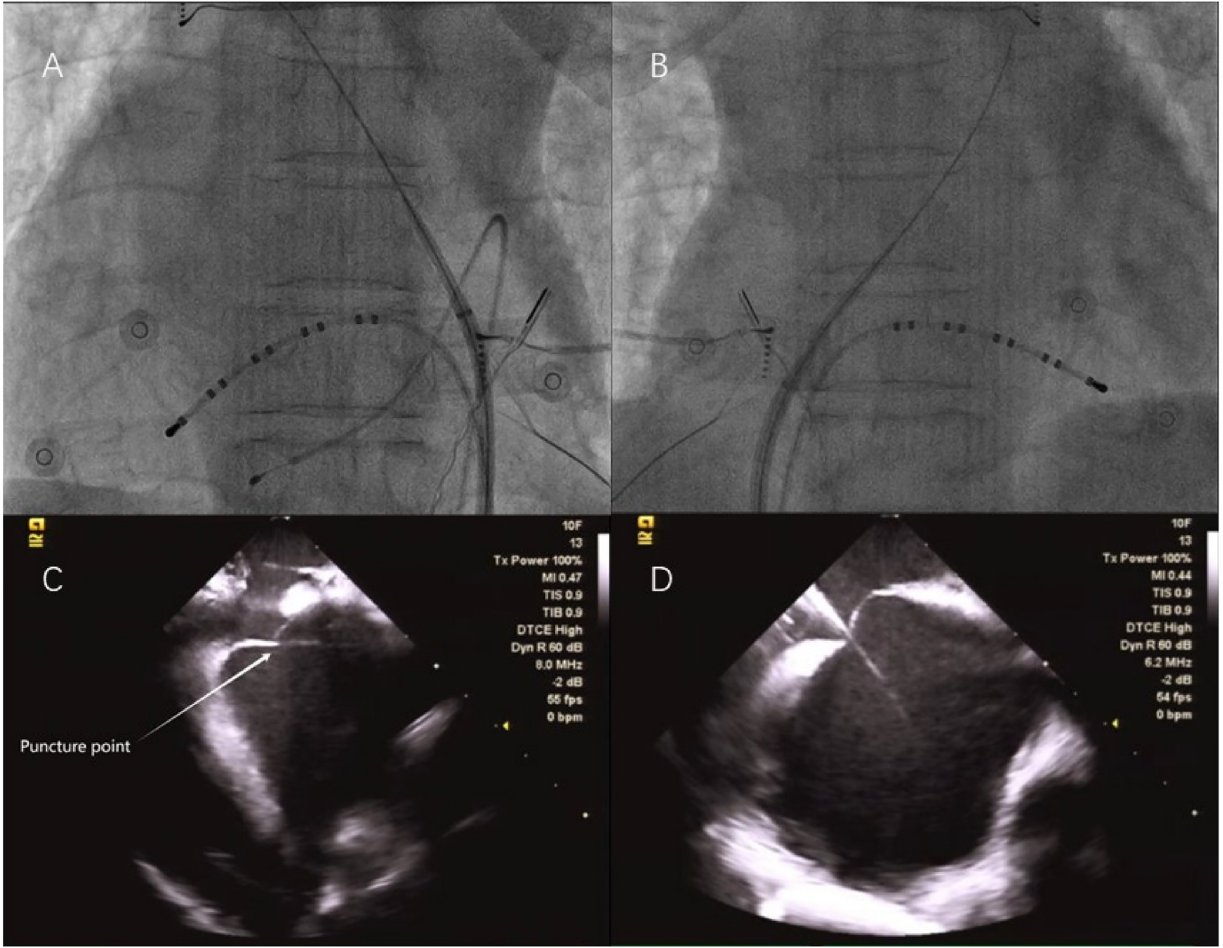
**(A)** X-ray image of the first transseptal (TS) puncture at position A–P; **(B)** X-ray image of the second TS puncture, taken at a 180° mirror image; **(C)** Intracardiac echocardiography (ICE) image of the first TS puncture; and **(D)** ICE image of the second TS puncture.

**Figure 4:**
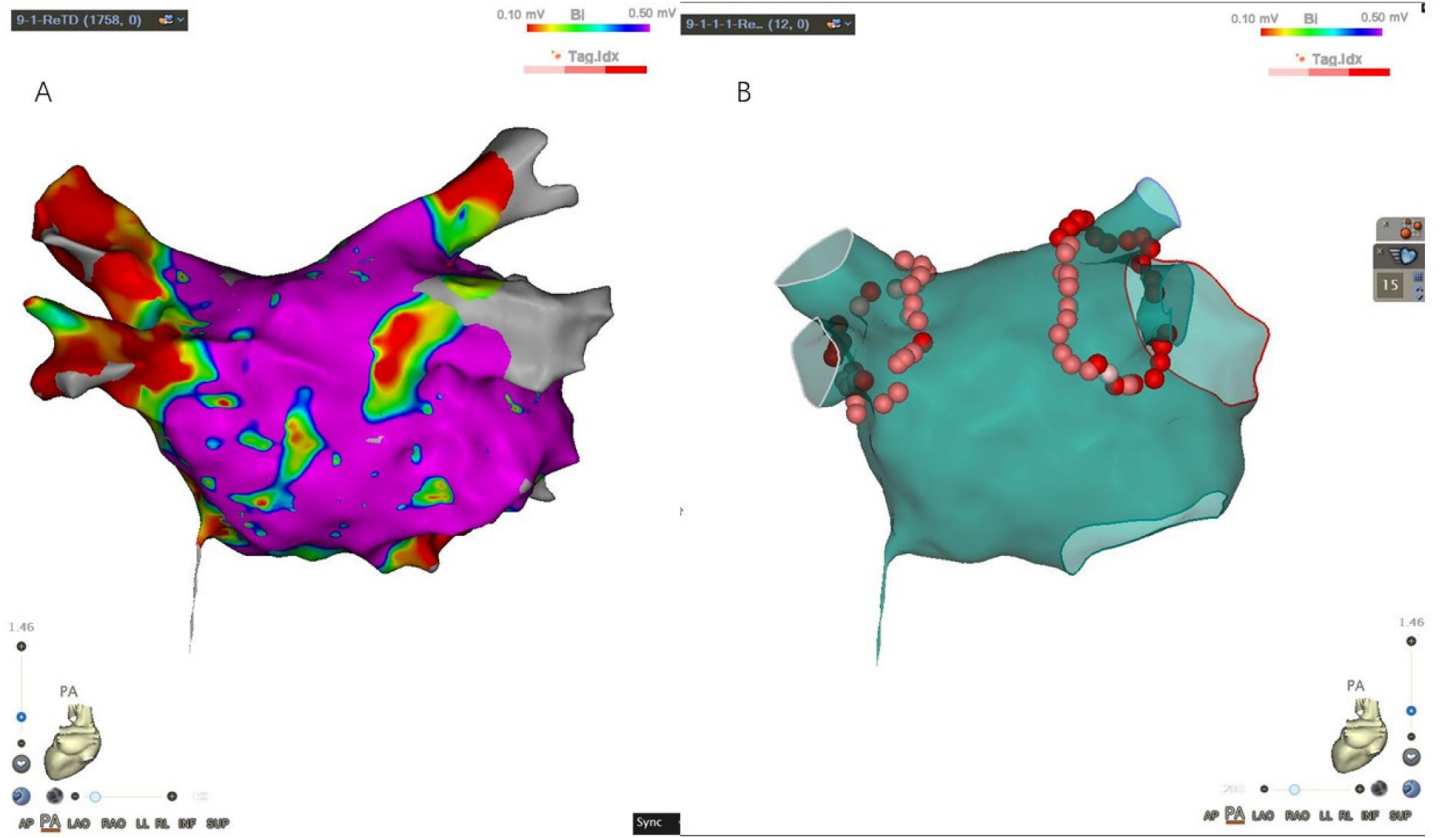
**(A)** Left atrial electroanatomical map during atrial fibrillation; **(B)** ablation map.

Given the patient's advanced age and dextrocardia, the heart structure was relatively complex. To ensure a safe “one-stop” procedure, we used ICE to exclude LAA thrombus, assist with puncture guidance, and monitor the pericardium. The ventricular electrode was advanced under fluoroscopy in an anteroposterior view, while the coronary sinus electrode was introduced under fluoroscopic guidance at a 45° right anterior oblique view. Despite thorough preparation, transseptal (TS) puncture was a challenging task. Fortunately, we completed it under ICE and x-ray guidance, with fluoroscopic angles opposite those typically used in standard TS punctures. The needle tip was directed toward the patient's leg at 10–11 o'clock, contrary to the usual 4–5 o'clock direction, with the puncture site confirmed under a 45° left anterior oblique view and ICE (Figure 3). A long guide wire was inserted in the right anterior oblique position to confirm entry into the left atrium (LA). Heparin (6,000 IU) was administered based on the patient's body weight, and the activated coagulation time was maintained at 250–300 s. A Pentaray electrode was used to construct the electroanatomical voltage mapping of the LA and pulmonary veins (Figure 4A). Bilateral pulmonary vein isolation was performed during AF (Figure 4B), and no pulmonary vein potentials were detected after a 30 min observation period.

**Figure 5:**
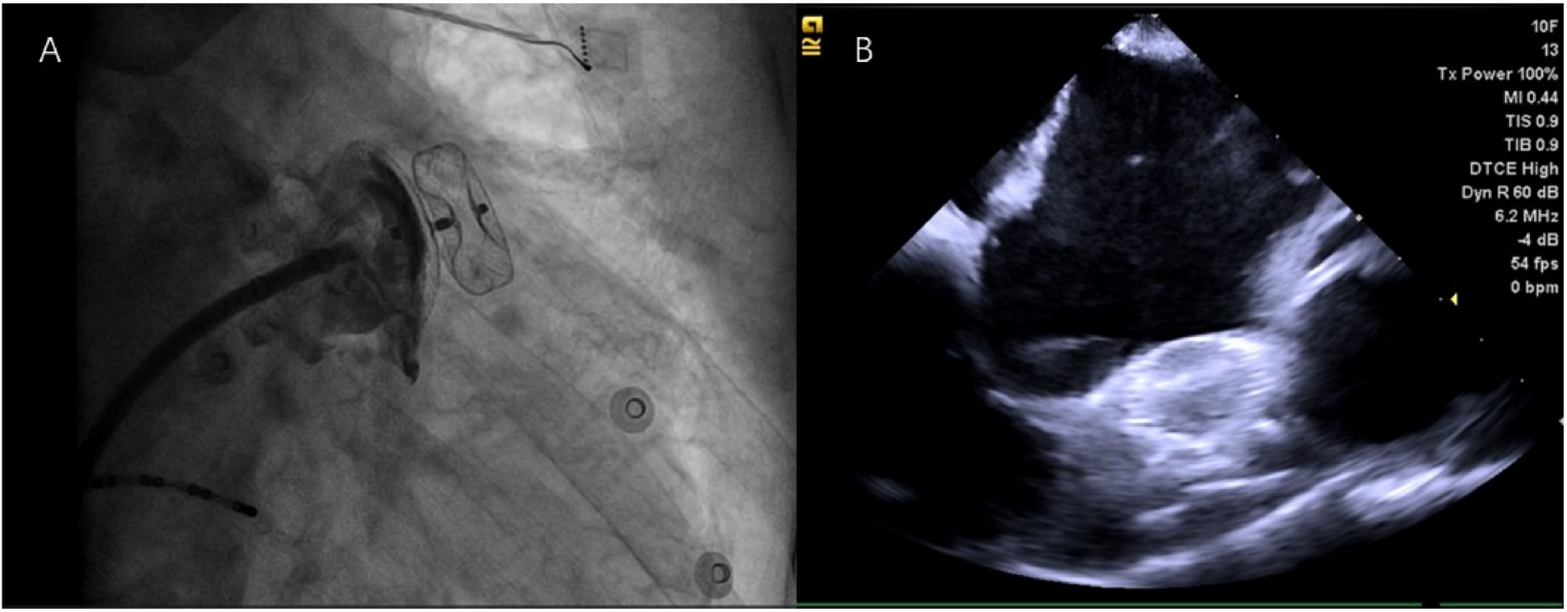
No peridevice leaks were detected after the procedure, as shown in x-ray **(A)** and intracardiac echocardiography **(B)**.

**Figure 6:**
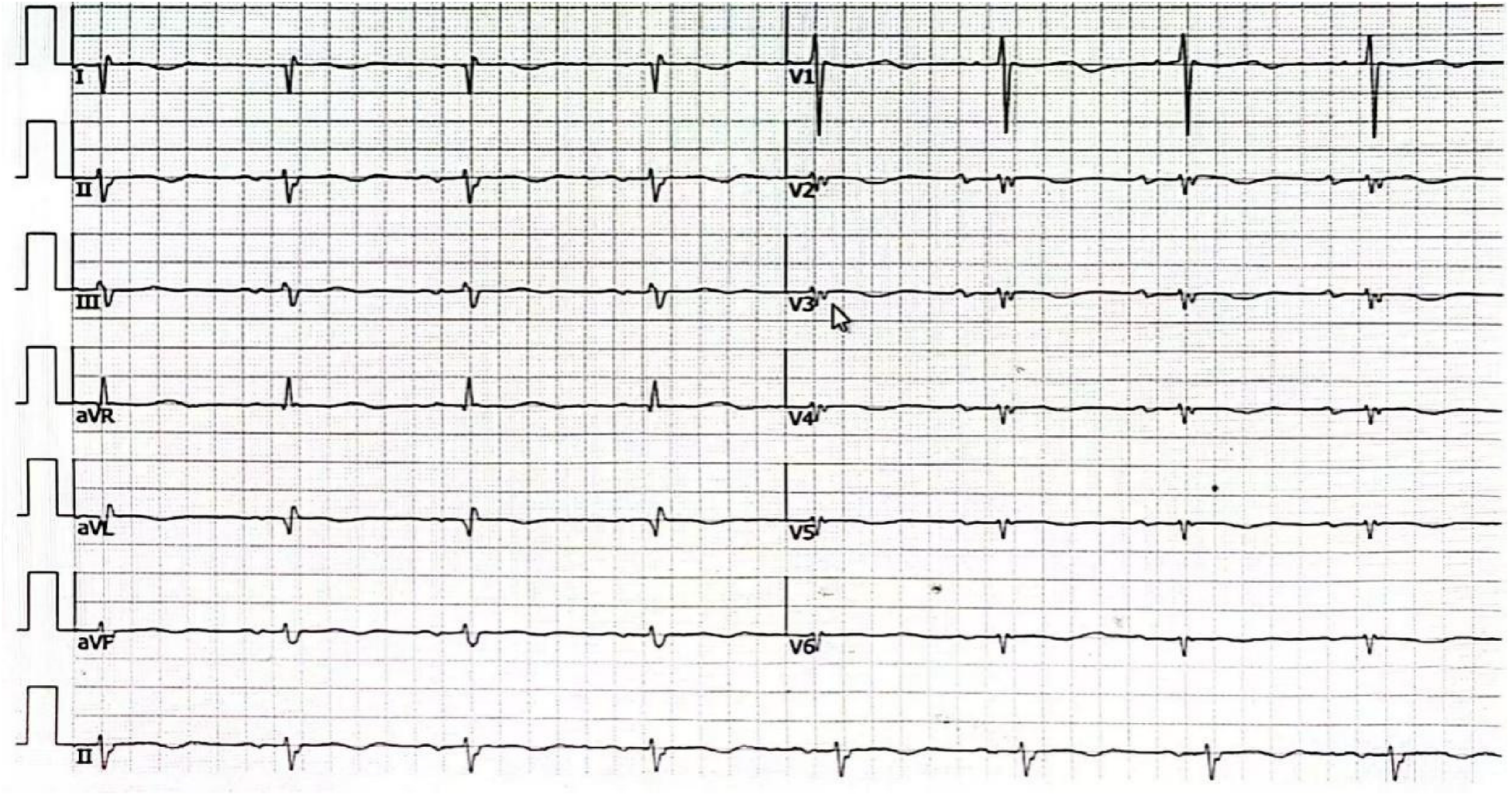
The electrocardiogram of the patient remained in sinus rhythm 3 months after the procedure.

After withdrawing the ablation catheter, we attempted to place the pig-tail catheter into the LAA along the TS sheath. However, due to the initial puncture site being backward and upward, we performed a second TS puncture. This second puncture differed in two key aspects: (1) the patient had an atrial septal aneurysm (ASA), which made it difficult to direct the Swartz sheath to the proper puncture site. Therefore, we used the VIZIGO bidirectional adjustable bent sheath; and (2) we adjusted the angiographic system settings to achieve a 180° mirror image (Figure 3). Though the process was challenging, the second puncture site was lower, facilitating subsequent LAAO. The LAA was angiographically evaluated, and its size was measured. A LAAO-I 28 device was selected and deployed under x-ray guidance. Both final angiographic and ICE results were excellent, with no peridevice leaks detected after the procedure (Figure 5). The patient continued to receive rivaroxaban (15 mg/day) and amiodarone (200 mg/day) after surgery. At the 3-month follow-up, the patient remained in sinus rhythm (Figure 6) with no postoperative complications. The oral anticoagulant therapy was discontinued, and the patient was switched to aspirin (100 mg/day) and clopidogrel (75 mg/day) for antiplatelet therapy (Table 1).

**Table 1:** A table displaying the timeline with relevant data from the episode of care.

2023.3.15	Cerebral infarction, CT: congenital total visceral inversion.
2023.3.15–2023.4.11	Oral anticoagulant (Rivaroxaban 15 mg/day).
2024.4.11	Recurrent cerebral infarction.
2024.5.29	“One-stop” surgery was performed in our hospital.
2024.5.29–2024.9.1	Rivaroxaban 15 mg/day.
2024.9.1–Present	Aspirin 100 mg/day and clopidogrel bisulfate 75 mg/day.

## Discussion

As far as we know, this is the first reported case of successful “one-stop” surgery in a patient with dextrocardia. Despite encountering some unexpected challenges, the procedure proceeded smoothly and safely, ultimately delivering significant benefits to the patient.

The cardiac anatomy of patients with dextrocardia differs from that of individuals with normal heart positioning, which undoubtedly increases the difficulty of the surgery. According to previous literature (3–5), adjusting the settings of the angiographic system to achieve a 180° mirror image may be highly beneficial to the success of the operation.

ICE plays two key roles during the procedure: (1) detecting the pericardium by advancing the ICE catheter through the femoral vein into the middle of the right atrium to establish the “Home View”, then withdrawing it to the “Euclistosis ridge” view of the lower right atrium. The catheter is then bent forward across the tricuspid valve into the right ventricle and rotated counterclockwise after slightly easing the bend, and (2) assisting with TS puncture by slightly advancing the ICE catheter from the “Home View” and rotating it counterclockwise by approximately 90° to visualize the fossa ovalis.

Accurate localization of the TS puncture site is crucial for performing “one-stop” surgery in patients with dextrocardia and AF. Given the challenges associated with TS puncture in these cases, intraoperative ultrasound imaging is essential, particularly when dealing with complex atrial septal anatomies (e.g., thickened interatrial septum and ASA). The VIZIGO bidirectional adjustable bent sheath can assist in the puncture. Moreover, a lower TS puncture site is critical for patients with dextrocardia to complete LAAO successfully.

This case, however, has certain limitations and deficiencies. For example, (1) the postoperative follow-up period is relatively short, and (2) this is a single case, which makes it difficult to generalize the findings to a broader population.

## Data Availability

The original contributions presented in the study are included in the article/Supplementary Material, further inquiries can be directed to the corresponding authors.
